# Overview of Polyethylene Terephthalate Foils Patterned Using 10 MeV Carbon Ions for Realization of Micromembranes

**DOI:** 10.3390/mi14020284

**Published:** 2023-01-22

**Authors:** Mariapompea Cutroneo, Vladimir Havranek, Anna Mackova, Petr Malinsky, Romana Miksova, Giovanni Ceccio, Lucio Ando’, Alena Michalcova

**Affiliations:** 1 Nuclear Physics Institute, The Czech Academy of Sciences (CAS), 25068 Rez, Czech Republic; 2 Department of Physics, Faculty of Science, University of J. E. Purkyně, Pasteurova 3544/1, 40096 Ústí nad Labem, Czech Republic; 3 National Institute of Nuclear Physics-INFN, Sezione di Catania, Via S. Sofia 64, 95123 Catania, Italy; 4 Department of Metals and Corrosion Engineering, University of Chemistry and Technology, 16628 Prague, Czech Republic

**Keywords:** polyethylene terephthalate, ion lithography, membrane, STIM, simulation

## Abstract

Polymer membranes are conventionally prepared using high-energy particles from radioactive decay or by the bombardment of hundreds of MeVs energy ions. In both circumstances, tracks of damage are produced by particles/ions passing through the polymer, and successively, the damaged material is removed by chemical etching to create narrow pores. This process ensures nanosized pore diameter but with random placement, leading to non-uniform local pore density and low membrane porosity, which is necessary to reduce the risk of their overlapping. The present study is focused on the use of polyethylene terephthalate (PET) foils irradiated by 10.0 MeV carbon ions, easily achievable with ordinary ion accelerators. The ion irradiation conditions and the chemical etching conditions were monitored to obtain customized pore locations without pore overlapping in PET. The quality, shape, and size of the pores generated in the micromembranes can have a large impact on their applicability. In this view, the Scanning Transmission Ion Microscopy coupled with a computer code created in our laboratory was implemented to acquire new visual and quantitative insights on fabricated membranes.

## 1. Introduction

Over the last 80 years, since the first polymer made of cellulose appeared at the International Exhibition in London, polymers have attracted the attention of the scientific community for their versatility and low cost. Ionizing radiation, including lasers [1], electron beams, ions, and inclusion of fillers, have been applied to modify the composition [2], structure, surface [3], optical [4], and electrical properties as well as density and porosity [5] of based polymers. 

In addition to these techniques, swift heavy ions are routinely employed for the creation of membranes set out for liquid, gas filtration, cell trapping, and packaging. 

It is established that energetic ions have sufficient energy and mass to penetrate solids in a straight path and produce a continuous damage line, named a latent track. The material in the ion tracks is preferentially removed by chemical etching resulting in arrays of nearly parallel hollow cylinders with diameters less than 10 nm. The track etch ratio, which is the ratio between the track etch rate, and the bulk etch rate, is the most important parameter determining the quality of the pores. The etching process is connected to the energy deposition density of the ion along the track, the radiation sensitivity of the material, the storage conditions of the ion-irradiated material before the chemical etching, and the etchant type, concentration, and temperature.

Inside the ion-irradiated polymer, it is possible to discriminate two regions, the track core and the halo track. The halo is created due to secondary electrons and mobile radiolysis products at radial distances higher than the track core radius.

Typically, the dominant cross-linking in the halo induces an etching rate outside the track core lower than in a virgin polymer [6]. According to available data, the latent track halo has not been measurable by any microscopy method. The existence of the latent track halo is suggested by the spatial distribution of monomer grafting in the polymers [7] irradiated by ions and in the shape of etched nanopores [8]. The sharp variation of the pore size [9], known as the bottleneck, can be responsible for constrictive effects, which have been shown to be a good predictor of diffusive transport through porous materials by Stenzel et al. [10].

Generally, heavier ions lead to well-defined ion tracks, and the ideal conditions seem to be reached for Kr or Xe ions [11,12] at energies up to about 20 MeV/nucleon.

Following the attention attracted by the wide applicability in material science, nanotechnology, and biophysics of the membranes, there is a clear demand for exploring routes to improve accessibility and cost in their production. 

The facilities in Brookhaven, Dubna, Louvain-la-Neuve and Berlin already provide high ion energy per nucleon, low fluence, large scanning area, pore diameters ranging from 0.01 to tens of micrometers and pore densities from about 10^4^ to 10^9^ cm^−2^. However, the use of more common accelerators delivering ions with energies ranging between hundreds of keVs and tens of MeVs [13] would be advantageous and cheaper. 

In the present study, the use of ion lithography [14] is proposed to fabricate membranes on polyethylene terephthalate (PET) foil with customized patterns [15], without the use of any mask, with good control of the irradiated area, using 10 MeV carbon ions conventionally generated by a 3 MV Tandetron accelerator. A higher surface density and negligible overlapping can be demonstrated in the resulting membranes over other approaches. Further benefits are ascribable to the good membrane reproducibility and the customized pore locations suitable for microfluidics and organ-on-chip applications. In addition, since the pore geometry, size and shape strongly influence the membrane properties, the complex damage structure [16] in the ion tracks, which can give insights into the pore size, shape, and connectivity, has been determined by the combination of Scanning Transmission ion Microscopy [15] and the TrackHH code created in our laboratory. 

The effectivity of the proposed approach for the characterization of the produced membranes comes from the fact that most of the existing methods cannot precisely capture tortuous paths unless relatively straight with well-connected pore networks and very thin foil thickness.

## 2. Materials and Methods

### 2.1. Ion Lithography

**Figure 1:**
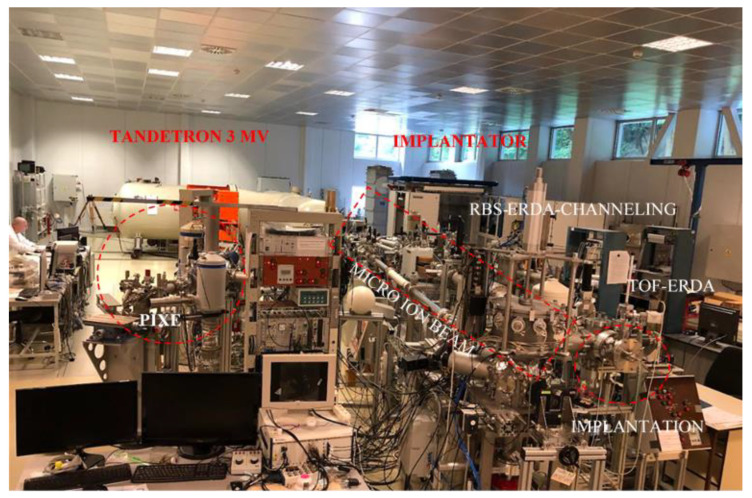
Picture of the Tandetron lab of the NPI in the Czech Republic.

PET foils 6 μm thick and with a density of about 1.397 g/cm^3^ were purchased from Sigma Aldrich. Pieces of PET foil with an area of 1.5 × 1.5 cm^2^ have been irradiated by an ion microbeam system at the Tandetron lab [17] in the Czech Republic (see Figure 1). In accordance with SRIM [18], carbon ions with an energy of 10 MeV focused to a beam spot size of 1 μm and current of 1.2 pA have been generated with a duoplasmatron ion source [17] and selected to obtain a proper projected range of about 11 μm, with electronic-stopping power of about 970 keV/μm and nuclear-stopping power of about 0.980 keV/μm calculated for our sample’s composition and density. 

The projected range of the employed ions was higher than the PET thickness to ensure they passed through the foil, inducing cylindrical damage orthogonally to the foil thickness.

Typically, multiMeV or a few MeV ions delivered toward a polymer pass through it, generating latent ion tracks along their paths [19]. 

The PET foils have been assembled in customized holders to assist the ion irradiation by 10 MeV C^4+^ ions in a vacuum of about 10^−6^ mbar at fluences of 3 × 10^10^ ions/cm^2^, 1 × 10^11^ ions/cm^2^ and 1 × 10^12^ ions/cm^2^. The adopted ion fluences have been evaluated before and after the ion irradiation by measuring the beam current with the Faraday cup, knowing the exposed irradiation area in the pattern and the exposure time. 

**Figure 2:**
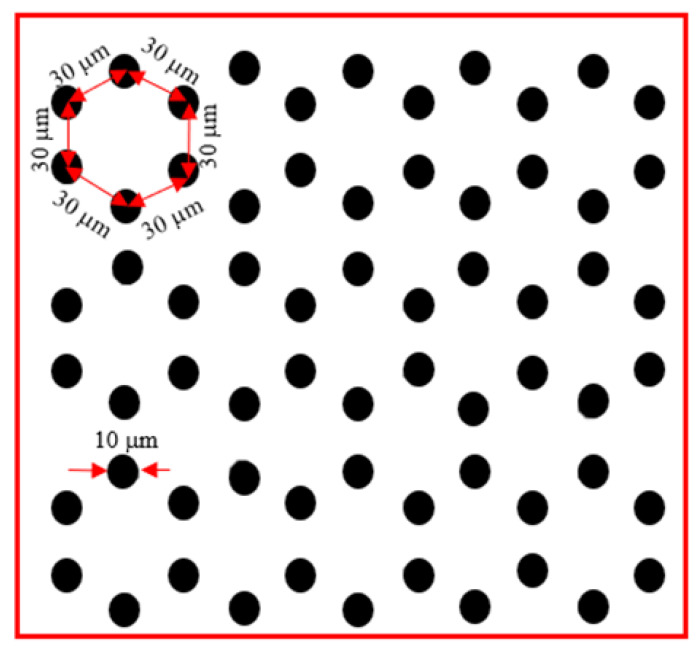
Sketch of the selected pattern to be irradiated on the PET foils by the micro ion beam. (A color version of this figure can be viewed online).

Ion lithography is a well-established technique where ions hitting the sample surface in selected areas induce the transfer of high-fidelity patterns to the surface. The in-house software, Scan5.vi, written in LabView code, has been employed to render the chosen drawing of the pattern (see Figure 2) on the foil by ion irradiation. It guides the structuring of the PET foils through control of the x-y scanning of the focused ion beam over the patterning and through control of beam blanking while skipping the beam to the next irradiation position. The fluency of ions on the pattern has been evaluated, taking into account the scan velocity and the number of scans. The irradiation time per pixel lasted 158 μs/pixel, 178 μs/pixel, and 533 μs/pixel corresponding to a total scan time of 108 s, 331 s, and 420 s to provide a total scan fluence of 3 × 10^10^, 1 × 10^11^, and 1 × 10^12^ ions/cm^2^.

Figure 2 shows the pattern designed to be reproduced on PET foils and saved in a monochrome bitmap to be recognized by the adopted software.

The selected dose was controlled by scan velocity and the number of loops. The ion beam was guided vertically, from top to bottom and back again, over the pattern area of 1024 μm × 1024 μm, setting the blanking time to 10.1 ms and the loop number to 3.

### 2.2. Chemical Etching

The chemical etching in an aqueous solution of NaOH with a concentration of 1 N and temperature of 50 °C in the air is typically employed to dissolve the material inside the track, obtaining shallow channels. 

The 6 μm PET foils irradiated at fluences from 3 × 10^10^ ions/cm^2^, 1 × 10^11^ ions/cm^2^, and 1 × 10^12^ ions/cm^2^ have been drenched in 1N (NaOH) solution at 50 ± 0.5 °C for 80 min (min) while the additional 6 μm thick PET foil irradiated at a fluence of 1 × 10^12^ ions/cm^2^ has been soaked for 150 min.

The chemical etching is performed at 50 °C; otherwise, at temperatures close to the glass transition, the rearrangement of the polymer on a molecular scale recovering the ion track to a pristine-like condition would be accelerated. The etched PET foils were washed in water under agitation to disable the etching process.

### 2.3. Scanning Transmission Ion Microscopy

Scanning Transmission Ion Microscopy (STIM) has been used to address the study of the material removal inside the latent track as well as the quality of the realized pores without altering the investigated material. The STIM measures the material cross-section through the evaluation of the lateral energy loss through the sample. This energy loss corresponds to the local thickness of the foil, taking into account that the energy loss for unit thickness for a specific material is noted. The energy loss of the ions traversing the foil denotes the depth of the material eliminated from the pores and if the pores have been broken through. Helium ions have been focused to a beam spot size better than 0.5 μm and scanned on the sample, as detailed in Ref. [20], for the evaluation of the etched pore size [15]. The beam current was set to about 2 Kparticles/sec by direct reading from the STIM Si PIN diode detector.

The PET foils chemically etched for 80 min have been analyzed using 3.5 MeV energy of He^2+^, while the PET foil chemically etched for 150 min has been investigated using 3.0 MeV energy of He^2+^. The PET penetration of the helium ions with energies of 3.0 MeV and 3.5 MeV leads to a depth of 13.8 μm and 17.0 μm, respectively, with energy loss but without inducing degradation along their trajectory attributable to the very low fluences (~10^6^–10^8^ ions/cm^2^) adopted for STIM analysis. The ion energy loss is strongly affected by the alteration of density along the path in the foil. Consequently, the energy spectrum reflects the foil density inhomogeneities. In 10 min, 2-dimensional maps of each pattern were captured in scanning windows of 500 μm × 500 μm whereas to retrieve the pore size, a more detailed scan size of 75 μm × 75 μm was employed. Both energy spectra and elemental maps of regions and planes of interest also for each individual pore have been carried out using the OM-DAQ software from Oxford Microbeams (OM)-UK [21].

### 2.4. Simulation Program for Pore-Shape Evaluation

Back in the 1990s, the TOM (shortcut for tomography) code [22] was created for the investigation of the radial structure of latent tracks using the energy spectrum of the alpha particles emitted by 241Am α-source, with 5.486 MeV main alpha energy line crossing a membrane. Therefore, the spectra evaluation was based on the use of the energy range of alpha particles emitted by 241Am (that is 85% of 5486 keV, 13% of 5443 keV, and about 2% of 5388 keV). 

Presently, the monoenergetic ions transmitted through a membrane and monitored by a Si PIN diode detector (S14605- Hamamatsu, Hamamatsu Photonics, Shizuoka, Japan) located behind the investigated foil are influenced by several parameters (foil composition and thickness, density variation, inhomogeneities), making the use of TOM challenging. The proposed TrackHH code based on the Monte Carlo simulation is the corrected version of the previous TOM. The modeling of the pore size and shape is obtained through the comparison of the experimental and the simulated energy spectra. The spectra are computed using a very low ion beam divergence and almost 0° incidence angle and use the following free parameters: thickness, density, inhomogeneities and structure of the used foil, number of tracks formed in the foil, and pore geometry. 

## 3. Results

**Figure 3:**
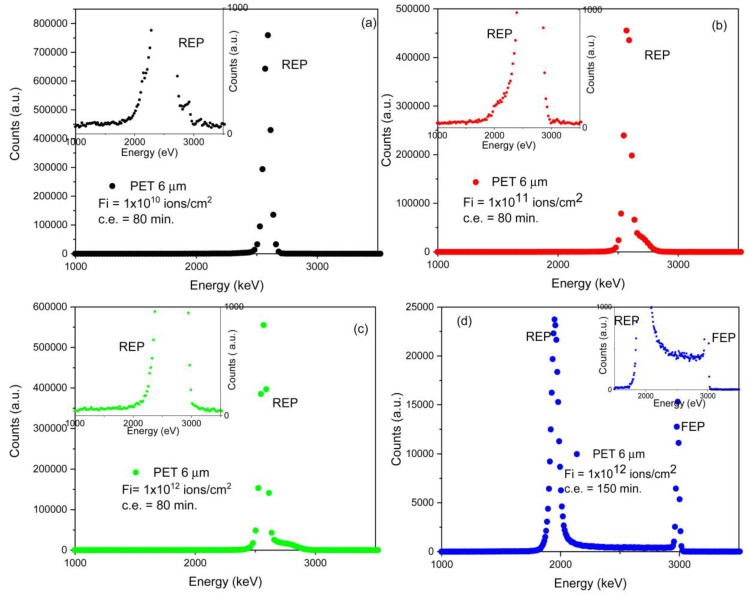
Spectra of the energy loss of He^2+^ ions 3.5 MeV for PET foils irradiated at fluences of 1 × 10^10^ ions/cm^2^ (**a**), 1 × 10^11^ ions/cm^2^ (**b**), 1 × 10^12^ ions/cm^2^ (**c**) and chemically etched for 80 min. The spectrum of energy loss of He^2+^ ions 3.0 MeV for PET foils irradiated at a fluence of 1 × 10^12^ ions/cm^2^ (**d**) and chemically etched for 150 min. In the inserts are shown the magnified spectra.

The STIM analysis provided the energy spectrum of transmitted He^2+^ ions at 3.5 MeV (see Figure 3a–c) and 3.0 MeV (see Figure 3d) energies hitting the treated (ion irradiated and chemically etched) PET foils and recorded by the Si PIN diode detector.

In Figure 3a–c, the energy spectra are referable to the 6 μm PET foils irradiated at 3 × 10^10^ ions/cm^2^ (see Figure 3a), 1 × 10^11^ ions/cm^2^ (Figure 3b), and 1 × 10^12^ ions/cm^2^ (Figure 3c) chemically etched for 80 min while in Figure 3d the energy spectrum relates to the PET foil irradiated at 1 × 10^12^ ions/cm^2^ followed by 150 min of chemical etching.

In Figure 3d, the Reduced Energy Peak (REP) is displayed, ascribable to the particles passing through the full thickness of the foils (i.e., virgin foil), and the Full Energy Peak (FEP), as a result of the particles transmitted without energy loss through a shallow pore (i.e., etched pore). In Figure 3a–c, only the REP is displayed, indicating that the adopted condition of the ion irradiation, etching time, and composition have not been able to induce the breaching of the pores.

The broadening of the REP energy peak is attributable to the straggling and multiple scattering of the particles.

The shape of the energy region between the REP and FEP peaks depends on the partial energy loss and leads to changes in the spatial form and size of the pores. 

Typically, the penetration of the matter by charged particles induces ion deceleration owing to the electronic (Se) and nuclear (Sn) stopping powers. To evaluate the energy loss mechanism indicated as linear energy transfer (LET) [23], SRIM code [18] has been employed assuming the PET composition (H = 36.4%, C = 45.3% and O = 18.3%) and density (1.397 g/cm^3^) provided by the selling firm. The prevailing contribution seems to be the electronic stopping which generally leads to atom ionization, electron excitation, and the creation of free radicals and free chemical bonds. The track etching rate decreases with decreasing ion energy, and in our case, the rate was sufficiently constant because 10 MeV carbon ions have a constant stopping power along their path in a 6 μm PET foil. Generally, at a constant track etching rate, the expected pore shape is conical (upon asymmetric etching [24]), double-cone or cylindrical.

### 3.1. Scanning Transmission Ion Microscope Analysis

For the STIM analysis, 3.5 MeV and 3.0 MeV He^2+^ ions have been selected by SRIM code [18] to convert the energy loss evaluation in thickness using a Monte Carlo simulation. For PET, assuming its atomic composition of 36.4% H, 45.3% C, and 18.3% O with a bulk PET density of 1.39 g/cm^3^, the energy loss for the 3.5 MeV helium ions passing through a PET foil is about 150 keV/μm and for 3.0 MeV is about 165 keV/μm at the surface, and it slightly increases up to the depth at 6 μm.

**Figure 4:**
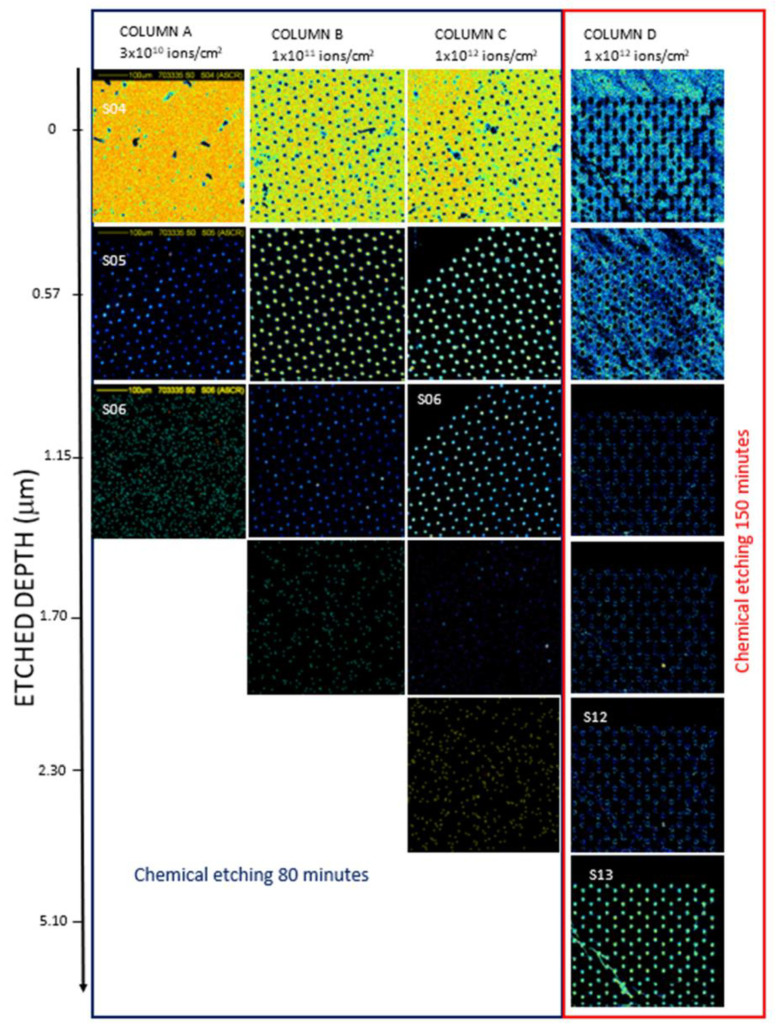
STIM images 500 μm × 500 μm scan size of the PET membrane obtained irradiating by C^4+^ ions at fluences of 3 × 10^10^ ions/cm^2^ column (**A**), 1 × 10^11^ column (**B**), 1 × 10^12^ column (**C**) chemically etched for 80 min and 1 × 10^12^ column (**D**) chemically etched for 150 min. (A color version of this figure can be viewed online).

**Figure 5:**
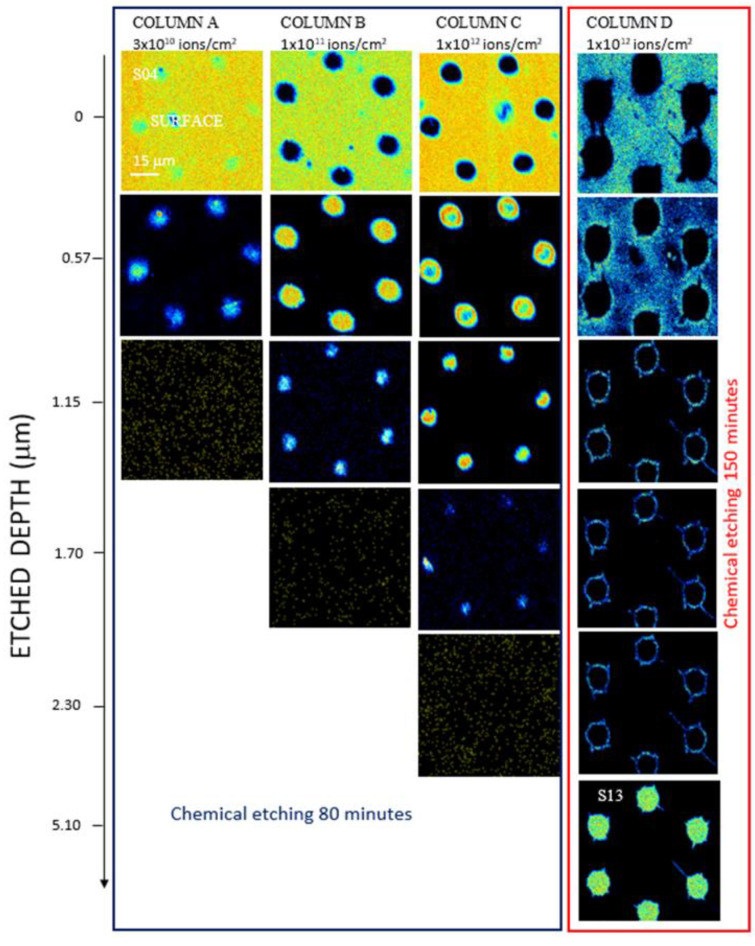
STIM images 75 μm × 75 μm scan size of the PET membrane obtained irradiating by C^4+^ ions at fluences of 3 × 10^10^ ions/cm^2^ column (**A**), 1 × 10^11^ column (**B**), 1 × 10^12^ column (**C**) and chemically etched for 80 min and 1 × 10^12^ column (**D**) chemically etched for 150 min. (A color version of this figure can be viewed online).

Considering the virtual energy loss windows in STIM energy spectra, the first STIM slice is ~0.6 μm thick, corresponding to the energy loss of nearly 132 keV. Figure 4 and Figure 5 show the density maps of the transversal plane of the foils for scan sizes of 500 μm × 500 μm and 75 μm × 75 μm, respectively, as a function of the depth. 

The PET foils implanted at fluences of 3 × 10^10^ ions/cm^2^ (column A), at 1x 10^11^ ions/cm^2^ (column B), 1x 10^12^ ions/cm^2^ (column C) all chemically etched for 80 min, and at 1x 10^12^ ions/cm^2^ and 150 min chemical etching time (column D). In all the maps, there are visible fragments and debris on the top layer of the foil attributable to the peeling due to the pressure in the etched track rising during the immersion in water after the chemical etching. Assuming that each slice of the sample is a window with thickness spread from the lower edge and the upper edge indicated in Figure 4 and Figure 5, the energy loss through each thickness window provides the value of the thickness window.

In Figure 4 and Figure 5, the light regions show the areas of particular depth, which is evidence of corresponding energy loss, whereas the dark regions correspond to all other thicknesses. The dark areas are not indicators of the impenetrability of the He ion beam but of the different energy loss at those locations. All the images are the outcome of transmitted ion images. The light areas in the top images (see Figure 4 and Figure 5) indicate the foil thickness. In Figure 5, COLUMN D, image S13 is the slice with no energy loss, so-called FEP (see energy spectrum in Figure 3), and it indicates the minimum diameter of the breached pores. In Figure 5, COLUMN D, images 3–5, only the bright edge of the circle is displayed because the formed track (pore length) is not perfectly cylindrical but slightly conical. 

Examining the maps displayed in the slices of PET for all the foils, the energy loss up to 2.30 μm, suggests that the pores have not been fully etched. The maps reveal the pores etched depth in PET irradiated at 3 × 10^10^ ions/cm^2^ (column A) as about 1.15 μm and at 1 × 10^11^ ions/cm^2^ (column B) at about 1.70 μm. The comparison between the PET foils irradiated at 1 × 10^12^ ions/cm^2^ (referring to columns C and D) reveals a pore etching of 2.30 μm depth in the former (column C) due to the lower chemical etching time (80 min).

The results indicate that the depth of the pores fabricated on PET foils by ion irradiation progressively increases with the ion fluence and, at the same ion fluence condition, increases with the chemical etching time. Figure 5 shows the details of the pore edges and the surface morphology of the polymeric foils at 75 μm × 75 μm scan size, considering one pattern as almost representative of the whole membrane.

Following Figure 5, it is revealed a decrease in the pore size from the top to the bottom of the foil and extracts the indicative shape of the pores obtained in the membrane. For the foils where the pores were not fully etched, only a rough estimation is possible up to the maximum obtained etched depth. The diameter of the pores in the slice corresponding at about 0.60 μm for 3 × 10^10^ ions/cm^2^ (column A) is about 11 μm, at a fluence of 1 × 10^11^ ions/cm^2^ (column B), the diameter of the pores is 12 μm, at a fluence of 1 × 10^12^ ions/cm^2^ (column C) the diameter of the pores is 14 μm. At a fluence of 1 × 10^12^ ions/cm^2^ (column D), corresponding to the only membrane fully etched, the surface of the pores exhibited an elliptical shape, and at about 0.6 μm depth, the diameter of the main axes was of about 22 μm and the depth of about 5.10 μm approximately indicating a cylindrical pore shape.

### 3.2. TrackHH Simulation Processing

**Figure 6:**
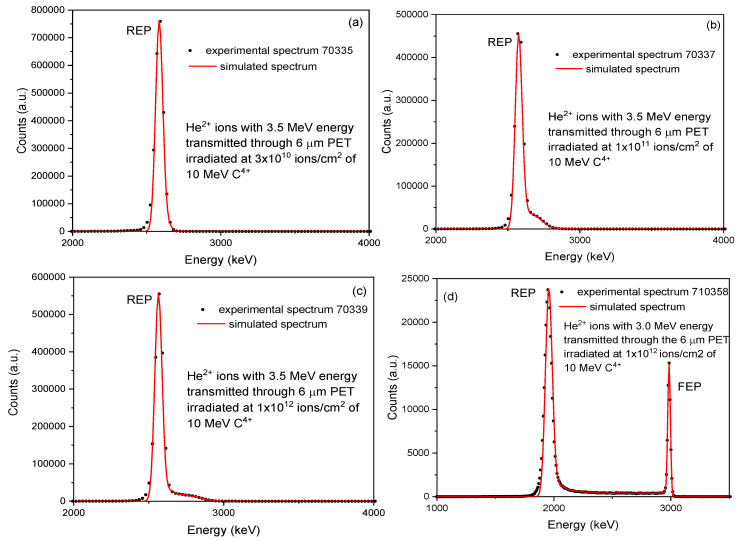
Comparison between the experimental and simulated spectra obtained by TrackHH code for the PET foils irradiated by ions. (A color version of this figure can be viewed online).

In accountancy of the correlation of the profile between REP and FEP and the shape and density of the pores, this area of the energy spectrum has been investigated for the evaluation of the pore shape [25] using the TrackHH simulation code [26]. The experimental spectra indicated by black dots (see Figure 6a–d) have been obtained from the ions transmitted through the PET foils and were recorded by the Si PIN diode.

The TrackHH simulation code is based on the use of the energy spectra of monoenergetic ions having random trajectories traversing the pores with different shapes and densities automatically acquired from files of ion energy loss, energy calibration of incident ions, the density of the used foil. The energy loss of transmitted ions has been evaluated point by point and subsequently converted into local thickness. The good agreement between the experimental and the simulated energy spectra revealed by the superimposition of experimental data (black dots) and simulated data (solid red lines) are displayed in Figure 6. This result has been obtained using 3.5 MeV energy (for Figure 6a–c), 3.0 MeV energy (for Figure 6d) of He^2+^ ions transmitted through a PET foil, taking into account the ion beam incidence angle of 0° and the divergence of the ion beam of about 0.14°.

The user, during the processing, adjusts the density of the tracks (i.e., REP and the FEP ratio) of about 0.8 × 10^5^, the pore shape file specifying the pore size at different depths and assuming the mean radius of the pore shape as circular.

**Figure 7:**
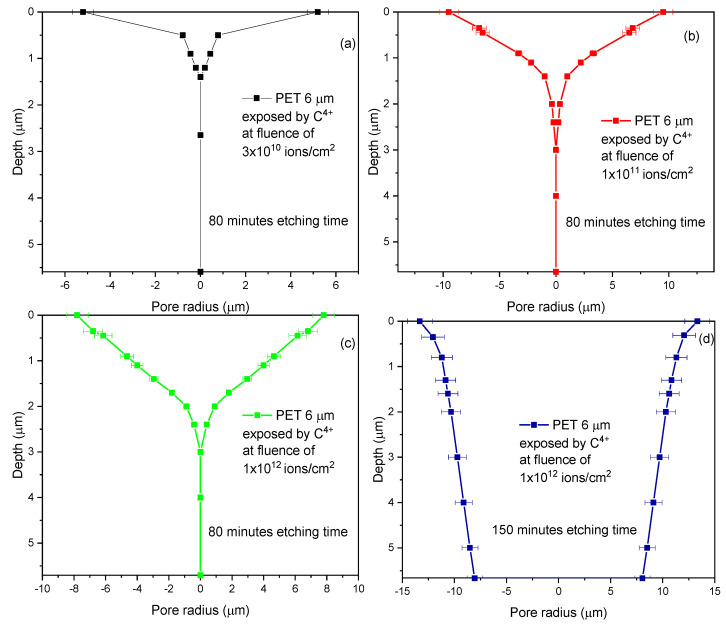
Model of the pores’ shape for the membranes obtained irradiating by C^4+^ ions at fluences of 3 × 10^10^ ions/cm^2^ column (**a**), 1 × 10^11^ ions/cm^2^ column (**b**), 1 × 10^12^ ions/cm^2^ column (**c**) chemically etched for 80 min and 1 × 10^12^ ions/cm^2^ column (**d**) chemically etched for 150 min. (A color version of this figure can be viewed online).

By linear interpolation of the distance between the upper surface foil at several discrete points (see Figure 7a–d), the local pore radius is calculated.

Figure 7 shows the simulations retrieved using the TrackHH software employed to study the area between FEP and REP, corresponding to the pore shape in the fabricated membranes. All the PET foils irradiated at ion fluences ranging between 3 × 10^10^ ions/cm^2^ and 1 × 10^12^ ions/cm^2^ and 80 min etching time membrane have been not fully etched as displayed in the epsilon shape of the simulations (see Figure 7a–c). Increasing the ion fluences, the pore is progressively opened, creating circular shapes with a maximum diameter of about 0.6 μm corresponding to about 10 μm (see Figure 7a), 11 μm (see Figure 7b), 13 μm (see Figure 7c) respectively. In the membrane produced by ion irradiation at a fluence of 1 × 10^12^ ions/cm^2^ and 150 min etching time, the pores are fully etched, show a diameter of about 20 μm, resemble a conical shape (see Figure 7d), and a height of about 5.60 μm in good agreement with the STIM analysis reported in Figure 4 and Figure 5.

The results suggest that the chemical attack in the ion-irradiated areas is more effective with the increase in the ion fluence and etching time in agreement with the TrackHH simulations and the literature.

## 4. Discussion

Despite the long tradition based on the use of energetic ions of the order of MeV/nucleon for the realization of polymeric membranes, possibly discouraging the exploration of the use of low-energy ions, the presented work was focused on the application of ion lithography for the realization of membranes. 

Over the undeniable advantages related to the use of ion lithography processing, the good control of the ion fluence and the high blanking time are crucial for the production of membranes enclosing pores in a chosen pattern. This technique minimizes the merging of the adjacent tracks and promotes the customization of the size and shape of the pores. A Tandetron accelerator, widely available in plenty of laboratories all over the world, was used to deliver carbon ions with an energy of 10 MeV focused by a micro ion beam system on a thin PET foil. The latent tracks formed by ion irradiation of the foils were converted into channels after chemical etching.

The improvement of the membrane efficiency, including the transport mechanism, the electrical conductivity, and the wettability, strongly depends on the control of the pore features during the fabrication. Pore size, shape, and composition are critical for application as catalytic membranes, gas or liquid sensors, or biomedical membranes. The geometry of the permanent changes in the ion-irradiated foil can be revealed by proper chemical etching conditions. Modifying the ion irradiation and the chemical etching is possible to obtain pores with desired geometry, size, and shape. It was observed that at an ion fluence of 1 × 10^12^ ions/cm^2^ and 150 min of chemical etching, the pores have been breached, obtaining a cylindric geometry.

The selective study of the optimal ion irradiation and chemical etching conditions to obtain good quality track-etched membranes has been supported by scanning transmission ion microscopy (STIM) coupled with the use of the TrackHH computer code for the evaluation of the presence of fully etched holes and their shape. Comparing the results obtained at ion irradiation of 1 × 10^12^ ions/cm^2^ and etching time of 80 and 150 min, it seems that the latter case represents the adequate protocol to obtain cylindric pores having a diameter of about 20 μm. In this view, it is reasonable to expect breached pores after 120 min, but the shape would probably be conical considering the evolution of the pore depth and shape at 3 × 10^10^, 1 × 10^11^, and 1 × 10^12^ ions/cm^2^, and 80 min of etching time.

To model the pore geometry, size, and thickness of the foil, the experimental and the simulated spectra have been compared. The proposed TrackHH cannot be precisely controlled, but its use combined with other analyses, such as STIM, provided consistent results. 

Both approaches are based on the analysis of the energy loss of monoenergetic ions traversing the membrane. 

The variation of the ion energy loss displayed in the spectra reveals the modification of the density in the ion tracks corresponding to fully or partially breached pores. Although the chemical etching severely affects the removal of the degraded material inside the latent track, it also attacks the surface of the foil, as confirmed by the reduction in the foil thickness from 6 μm (virgin) up to 5.6 μm (final foil after ion irradiation and chemical etching).

The pores formed in the 6 μm PET foils, irradiated at ion fluences ranging between 3 × 10^10^ ions/cm^2^ and 1 × 10^12^ ions/cm^2^ with 80 min etching time, were not broken through. In the beginning, the pores assumed a conical shape, which progressively refined into a cylinder form after 150 min of etching time. The size of the formed pores, when not fully etched, ranged between 10 μm and 13 μm. Conversely, at an ion fluence of 1 × 10^12^ ions/cm^2^ and 150 min etching time, the pores were fully etched and showed a cylindrical shape and diameter of about 20 μm. 

The joint application of STIM and TrackHH presented congruence in both the formed pore size and the value of the final foil thickness. The relative error in pore size can be estimated at about 10% by direct comparison between the value of the pore diameters evaluated by STIM and TrackHH code.

The fluctuations in the values of the relative stoichiometry of the material, sample thickness, and density, as well as etching area and etchant conductivity, have been assumed to be below 2%. The variation in the determination of the etching time is less than 1% while in etching temperature is within 0.5 °C. 

## Data Availability

Not applicable.
